# Microbiota prevents cholesterol loss from the body by regulating host gene expression in mice

**DOI:** 10.1038/srep10512

**Published:** 2015-05-27

**Authors:** Chun-Yan Zhong, Wei-Wei Sun, Yinyan Ma, Hongling Zhu, Pan Yang, Hong Wei, Ben-Hua Zeng, Qian Zhang, Yu Liu, Wen-Xia Li, Yixin Chen, Liqing Yu, Zhi-Yuan Song

**Affiliations:** 1 Department of Cardiology, Southwest Hospital, The Third Military Medical University, Chongqing, 400038, China; 2 Department of Animal and Avian Sciences, University of Maryland, 20746, USA; 3 Department of Laboratory Animal Science, College of Basic Medical Sciences, The Third Military Medical University, Chongqing, 400038, China

## Abstract

We have previously observed that knockout of Niemann-Pick C1-Like 1 (NPC1L1), a cholesterol transporter essential for intestinal cholesterol absorption, reduces the output of dry stool in mice. As the food intake remains unaltered in NPC1L1-knockout (L1-KO) mice, we hypothesized that NPC1L1 deficiency may alter the gut microbiome to reduce stool output. Consistently, here we demonstrate that the phyla of fecal microbiota differ substantially between L1-KO mice and their wild-type controls. Germ-free (GF) mice have reduced stool output. Inhibition of NPC1L1 by its inhibitor ezetimibe reduces stool output in specific pathogen-free (SPF), but not GF mice. In addition, we show that GF versus SPF mice have reduced intestinal absorption and increased fecal excretion of cholesterol, particularly after treatment with ezetimibe. This negative balance of cholesterol in GF mice is associated with reduced plasma and hepatic cholesterol, and likely caused by reduced expression of NPC1L1 and increased expression of ABCG5 and ABCG8 in small intestine. Expression levels of other genes in intestine and liver largely reflect a state of cholesterol depletion and a decrease in intestinal sensing of bile acids. Altogether, our findings reveal a broad role of microbiota in regulating whole-body cholesterol homeostasis and its response to a cholesterol-lowering drug, ezetimibe.

Trillions of bacteria live in our body, particularly in the gut lumen^1^. It has been demonstrated, mainly by using germ-free (GF) mice, that the gut microbiota critically regulates the pathogenesis of common metabolic diseases, such as central obesity, insulin resistance, type 2 diabetes, and nonalcoholic fatty liver disease^2^,^3^,^4^,^5^. Association studies suggest that the gut microbiota also impacts the development and progression of atherosclerotic cardiovascular diseases^6^,^7^. Increased blood low-density lipoprotein-cholesterol (LDL-C) is an independent risk factor for atherosclerosis^8^. Despite a key role of cholesterol balance in atherosclerotic cardiovascular diseases, only a few studies have shown the potential role of microbiota in regulating whole-body cholesterol homeostasis while focusing on other research topics, such as the high-fat diet (HFD)-induced insulin resistance^9^, the association between the gut microbiota and cholesterol metabolism in hamster^10^, and bile acid metabolism^11^.

In previous studies, we observed that the stool output is significantly reduced in Niemann-Pick C1-Like 1 (NPC1L1) gene knockout (L1-KO) mice on a Western type diet^12^, despite that L1-KO mice relative to their wild-type controls consume the similar amount of food^13^. NPC1L1 protein is essential for intestinal cholesterol absorption^14^ and is the molecular target of ezetimibe^15^,^16^, a cholesterol absorption inhibitor that has widely been prescribed to lower blood cholesterol in humans^17^. NPC1L1 is mainly expressed in small intestine across species^14^. Although human NPC1L1 is also expressed in other tissues^14^,^18^, the expression of mouse NPC1L1 appears to be restricted to the epithelium of small intestine and gallbladder^19^. As bacteria are very abundant in the gut lumen, we speculated that NPC1L1 deficiency may somehow alter the gut microbiome to reduce stool output, which may modulate the effect of NPC1L1 inhibition on cholesterol metabolism. In this study, we first sequenced 16S rRNA genes in fecal samples from L1-KO mice and their controls on both chow and a HFD and found that the gut microbiome differs significantly between the two genotypes and between the two diets. We then compared cholesterol homeostasis in both specific pathogen-free (SPF) mice and GF mice that were fed a Western diet with or without ezetimibe. The Western diet was chosen because its composition is similar to that in typical human diets in Western societies. We demonstrate that GF versus SPF mice have reduced stool output and that ezetimibe treatment reduces stool output in SPF, but not GF mice. Compared to SPF mice, GF have decreased intestinal cholesterol absorption and increased fecal cholesterol excretion, which is associated with reduced expression of NPC1L1 and increased expression of ATP-binding cassette transporter G5 and G8 (ABCG5 and ABCG8). Expression of other genes in intestine and liver largely reflects a negative balance of cholesterol in GF mice. Likely as a result, plasma and hepatic levels of cholesterol are decreased in GF mice. All of these changes are more dramatic in ezetimibe-treated GF mice than ezetimibe-treated SPF mice. Altogether, our findings suggest that lack of microbiota promotes cholesterol loss, at lest, in mice.

## Results

### Ezetimibe requires microbiota to reduce stool output

**Figure 1:**
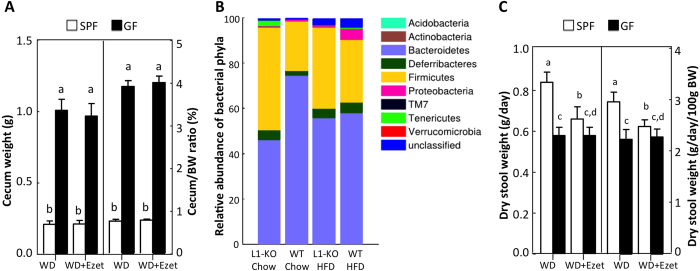
NPC1L1 and microbiota. ** (A)** Average wet weight of ceca and cecum-to-body weight (BW) ratios in SPF and GF mice treated with or without ezetimibe (n = 10(11). **(B)** Relative abundance of bacterial phyla in feces of L1-KO mice and their wild-type (WT) controls on a chow diet or a high fat diet (HFD) for 5 weeks (n = 4 for L1-KO/Chow, WT/Chow, and WT/HFD; n = 3 for L1-KO/HFD). Analysis was done by pyrosequencing of 16S rRNA genes. **(C)** Stool output in SPF and GF mice treated with or without ezetimibe (n = 10–11). The values with different small letters differ significantly (One-way ANOVA, p < 0.05). WD, Western diet; Ezet, ezetimibe.

To confirm the germ-free status of our GF mice, we measured weight of cecum because mega-cecum is a hallmark of GF mice^20^. We found that both cecum weight and cecum-to-body weight ratio were significantly increased by ~5-folds in GF mice relative to SPF mice, regardless of ezetimibe treatment (Fig. 1A). The germ-free status of our GF mice was also confirmed by the absence of bacteria-modified cholesterol, such as coprostanol, in the fecal samples from GF mice (data not shown) as well as reduced fat storage (Table S1), an observation seen in other GF mice^2^.

As we have previously shown that mice lacking NPC1L1 have reduced output of dry feces on a Western-type diet^12^ and consume similar amounts of food when compared to wild-type controls^13^, we speculated that NPC1L1 deficiency might alter the gut microbiota to reduce stool output. To determine whether NPC1L1 deficiency alters the gut microbiome, we performed 16S rRNA gene pyrosequencing of fecal samples collected from L1-KO mice and their controls on both chow and a HFD for 5 weeks. We observed that the relative abundance of Bacteroidetes was decreased while Firmicutes were increased in the gut of L1-KO mice compared to age-matched control wild-type mice housed in the same room (Fig. 1B), suggesting a substantial alteration of the gut microbiome in L1-KO mice. While the HFD feeding reduced Bacteroidetes and increased Firmicutes in the control wild-type mice, the opposite occurred in L1-KO mice (Fig. 1B). To determine whether lack of microbiota alters stool output, we collected feces for 3 days from SPF and age-matched GF mice fed a Western diet for 21 days and found that both daily dry stool weight and stool output (dry stool weight/100g BW/day) was significantly decreased in GF versus SPF mice (Fig. 1C). As expected, the NPC1L1 inhibitor ezetimibe reduced stool output in SPF, but not GF mice on the Western diet. This finding indicates that NPC1L1 inhibition requires microbiota, most likely the gut microbiota, to reduce stool output.

### Lack of microbiota reduces intestinal cholesterol absorption and promotes fecal neutral sterol excretion

**Figure 2:**
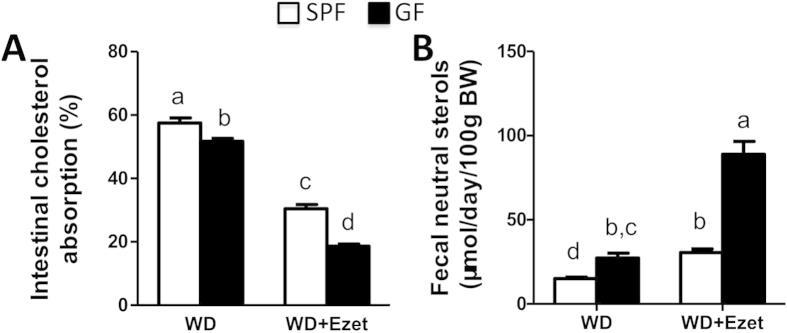
Cholesterol balance is altered in GF mice. ** (A)** Intestinal cholesterol absorption and **(B)** fecal neutral sterol excretion in SPF and GF mice on Western diet (n = 10-11). The values with different small letters differ significantly (One-way ANOVA, p < 0.05).

To determine how microbiota influences cholesterol balance, we measured intestinal cholesterol absorption and fecal neutral sterol excretion. We found that intestinal cholesterol absorption was lower in GF than SPF mice on Western diet (Fig. 2A). Ezetimibe treatment for 28 days reduced cholesterol absorption by ~47% and ~64% in SPF and GF mice, respectively. The reduction of intestinal cholesterol absorption associated with ezetimibe treatment was significantly larger in GF mice than SPF mice. Fecal neutral sterol excretion was significantly higher in GF mice than SPF mice (Fig. 2B). Ezetimibe treatment for 21 days significantly increased fecal neutral sterol excretion by ~104% and ~226% in SPF and GF mice, respectively. The increase in fecal neutral sterol excretion associated with ezetimibe treatment was significantly larger in GF mice than SPF mice.

### Lack of microbiota reduces biliary concentrations of cholesterol, phospholipids and bile acids

**Figure 3:**
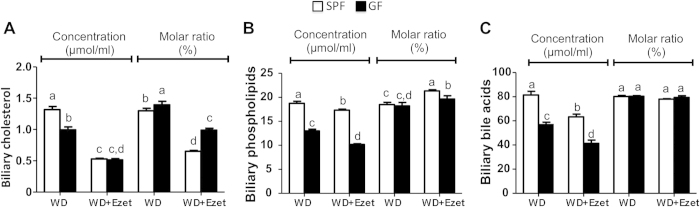
GF mice display reduced concentrations, but unaltered molar ratios of lipids in gallbladder. Gallbladder concentrations and molar ratios of cholesterol **(A)**, phospholipids **(B)** and bile acids **(C)** were analyzed in the mice fed a Western diet (WD) with or without ezetimibe (Ezet) for 38 days (n = 10-11). The values with different small letters differ significantly (One-way ANOVA, p < 0.05).

Biliary secretion is a major pathway for eliminating cholesterol from the body. Reduced absorption and increased fecal excretion of cholesterol may lead to decreased biliary secretion in GF mice. Indeed biliary concentrations of cholesterol were decreased in GF mice on Western diet (Fig. 3A). Ezetimibe treatment reduced biliary cholesterol concentrations in both SPF and GF mice likely due to inhibition of intestinal cholesterol absorption and reduced delivery of cholesterol to liver. Similar changes were also observed for biliary phospholipids and bile acids, though ezetimibe-associated changes were modest for these lipids (Fig. 3B,C). When molar ratios were calculated, it was found that the molar ratio of biliary cholesterol, but not phospholipids and bile acids, was increased (Fig. 3A–C), implying that GF mice may have increased cholesterol saturation index, a measure that is correlated with cholesterol gallstone formation^21^.

### Lack of microbiota reduces plasma lipids

**Figure 4:**
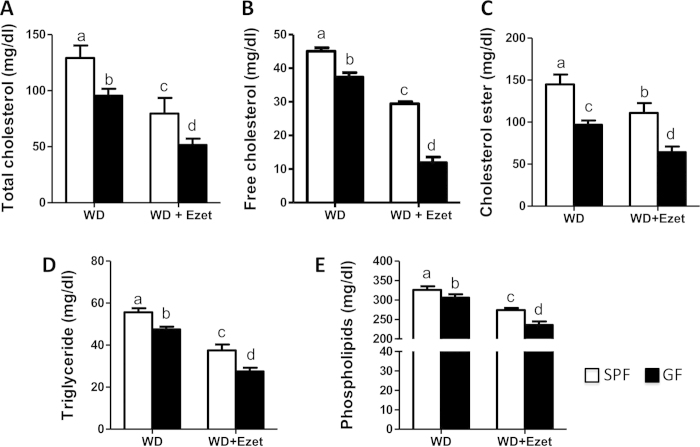
Plasma lipids are decreased in GF mice relative to SPF mice. Plasma concentrations of cholesterol **(A-C)**, triglycerides **(D)** and phospholipids **(E)** were measured in the mice fed a Western diet (WD) with or without ezetimibe (Ezet) for 38 days (n = 10-11). Cholesterol ester concentrations were calculated by multiplying the mass difference between total and free cholesterol by 1.67. The values with different small letters differ significantly (One-way ANOVA, p < 0.05).

To determine how altered cholesterol balance in GF mice affects blood lipids, we measured plasma concentrations of cholesterol, triglycerides and phospholipids. It was observed that all of these lipids were lower in GF mice than SPF mice (Fig. 4). Ezetimibe treatment had a greater effect on cholesterol and triglycerides in GF mice relative to SPF mice. After ezetimibe treatment, plasma total cholesterol (TC) was down by 46.1% in GF mice versus 36.4% in SPF mice, free cholesterol (FC) down by 64.0% in GF mice versus 47.1% in SPF mice; and cholesterol ester (CE) down by 33.1% in GF mice versus 27.9% in SPF mice. Plasma TG was down by 42% in GF mice, but only 33% in SPF mice. Ezetimibe treatment did not show differential effects on plasma phospholipids between GF and SPF mice (down by 17.2% in GF mice versus 15.9% in SPF mice).

### Lack of microbiota reduces hepatic lipids

**Figure 5:**
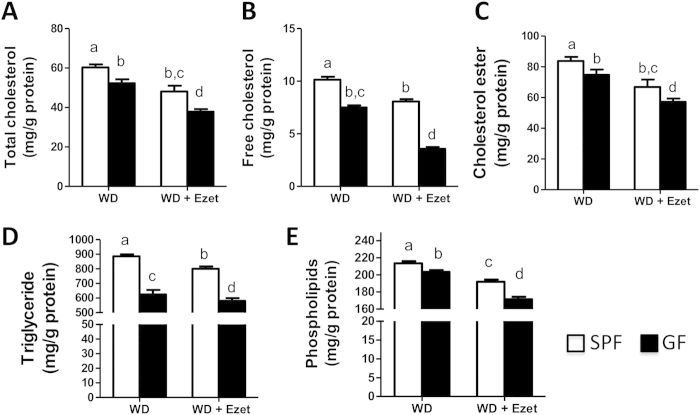
Hepatic lipids are reduced in GF mice compared to SPF mice. Hepatic contents of cholesterol **(A-C)**, triglycerides **(D)** and phospholipids **(E)** were measured in the mice fed a Western diet (WD) with or without ezetimibe (Ezet) for 38 days (n = 10-11). The content of cholesterol ester was calculated by multiplying the mass difference between total and free cholesterol by 1.67. The values with different small letters differ significantly (One-way ANOVA, p < 0.05).

Liver plays a central role in lipid metabolism. To determine how microbiota influences liver lipid homeostasis, hepatic contents of cholesterol, triglycerides and phospholipids were determined in GF and SPF mice. A significant reduction in hepatic contents of all lipids examined was observed in GF mice compared to SPF mice (Fig. 5). Ezetimibe treatment significantly reduced these lipids in both GF and SPF mice, and the reduction was greater in GF than SPF mice overall. Ezetimibe reduced hepatic total cholesterol by 27.7% in GF mice versus 20.3% in SPF mice (Fig. 5A); free cholesterol by 52.4% in GF mice relative to 20.5% in SPF mice (Fig. 5B); cholesterol esters by 23.6% in GF mice versus 20.3% in SPF mice (Fig. 5C); triglycerides by 15.7% in GF mice versus 9.7% in SPF mice (Fig. 5D); and phospholipids by 15.8% in GF mice versus 10.1% in SPF mice (Fig. 5E). The data of hepatic free cholesterol suggest that ezetimibe is likely more effective in lowering hepatic cholesterol when the microbiota is inhibited.

### Intestinal gene expression alterations suggest reduced uptake and increased excretion of cholesterol in GF mice

**Figure 6:**
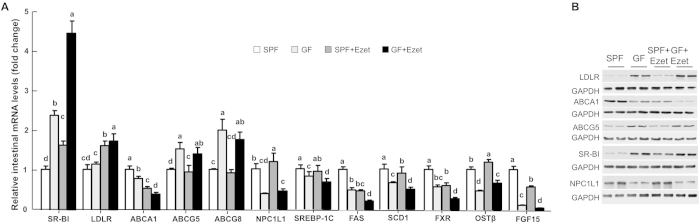
Intestinal expression levels of genes involved in lipid metabolism and bile acid sensing. The mice were fed a Western diet (WD) with or without ezetimibe (Ezet) for 38 days, followed by necropsy for tissue collection. **(A)** Relative mRNA levels analyzed by qPCR in the proximal second segment of small intestine that was divided into 5 equal segments (n = 6-7). GAPDH was used as an invariant control. The values with different small letters differ significantly (One-way ANOVA, p < 0.05). **(B)** Western blots of protein levels in the middle (third) segment of small intestine.

To explore potential mechanisms responsible for microbiota-associated changes in lipid homeostasis, we measured mRNA and protein levels of key genes relevant to lipid metabolism in the jejunum (Fig. 6). Scavenger receptor class B type I (SR-BI), a high-density lipoprotein (HDL) receptor^22^ that is also involved in cholesterol trafficking in enterocytes^23^, was significantly upregulated in GF versus SPF mice and ezetimibe treatment led to further increases in intestinal SR-BI at both mRNA (Fig. 6A) and protein (Fig. 6B) levels in both GF and SPF mice. LDL receptor (LDLR) mRNA levels displayed no substantial differences between GF and SPF mice, regardless of ezetimibe treatment. As expected, ezetimibe treatment raised LDLR mRNA levels in both groups likely due to upregulation of pathways in cholesterol synthesis in response to ezetimibe-induced inhibition of cholesterol uptake. Interestingly absence of microbiota increased protein levels of LDLR in the jejunum of GF mice compared to SPF mice, suggesting that microbiota may regulate LDLR expression via affecting LDLR mRNA and/or protein stability.

ABCA1 is a target gene of cholesterol sensor liver X receptor (LXR)^24^. Both mRNA and protein levels were reduced in GF mice and after ezetimibe treatment (Fig. 6). ABCG5 and ABCG8 are also targets of LXR^25^ and they form a heterodimer to transport cholesterol and noncholesterol sterols out of cells^26^,^27^,^28^,^29^,^30^. Their mRNA levels somehow went to the opposite direction of ABCA1, and were significantly increased in GF mice. So did ABCG5 protein. Due to antibody availability, we did not measure ABCG8 protein. NPC1L1 is essential for intestinal cholesterol absorption^31^. Both mRNA and protein levels of NPC1L1 were downregulated in the jejunum when microbiota was absent, implying that microbiota may promote intestinal cholesterol absorption by sustaining intestinal NPC1L1 expression.

Intestinal expression levels of lipogenic genes, including sterol regulatory element-binding protein-1c (SREBP-1c), fatty acid synthase (FAS) and stearoyl-CoA desaturase-1 (SCD-1), were lower in GF mice than SPF mice (Fig. 6A), which may reflect an overall low cholesterol content in intestinal cell because SREBP-1c is a target of cholesterol sensor LXR^32^ and activation of SREBP-1c increases expression of lipogenic genes including FAS and SCD-1^33^. An intriguing finding in comparing intestinal gene expression between GF and SPF mice was the observation that genes activated by bile acids, including a nuclear receptor farnesoid X receptor (FXR)^34^,^35^, organic solute and steroid transporter (Ost)-β^36^,^37^,^38^, and fibroblast growth factor (FGF)-15^39^ were significantly downregulated in GF mice (Fig. 6A). Although genes related to bile acid transport and sensing are normally examined in ilea where bile acids are reabsorbed, they are readily detectable in the middle segment of small intestine. This finding warrants further detailed studies of bile acid metabolism in GF mice.

### Hepatic gene expression alterations are consistent with reduced delivery of cholesterol to liver in GF miche

**Figure 7:**
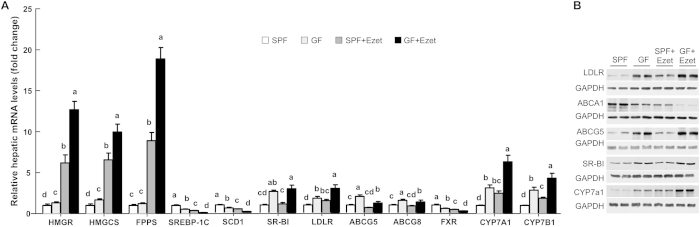
Hepatic expression levels of genes involved in lipid metabolism and bile acid metabolism. The mice were fed a Western diet (WD) with or without ezetimibe (Ezet) for 38 days, followed by necropsy for tissue collection. **(A)** Relative mRNA levels analyzed by qPCR (n = 6-7). GAPDH was used as an invariant control. The values with different small letters differ significantly (One-way ANOVA, p < 0.05). **(B)** Western blots of hepatic proteins.

To determine if hepatic lipid metabolism reflects reduced absorption and increased excretion of cholesterol in GF mice, we measured mRNA and protein levels of genes related to lipid synthesis and transport (Fig. 7). Consistent with reduced availability of cholesterol, hepatic mRNAs for 3-hydroxy-3-methyl-glutaryl-CoA (HMG-CoA) reductase (HMGCR, the rate-limiting enzyme in cholesterol biosynthesis), HMG-CoA synthase (HMGCS), and farnesyl diphosphate synthase (FPPS) were all upregulated in GF mice. Ezetimibe further increased expression of these genes in both SPF and GF mice with a much greater increase seen in GF mice. LXR-sensitive genes, ABCA1, SREBP-1c and SCD-1 were downregulated with a greater reduction in ezetimibe-treated GF mice (Fig. 7A,B). Liver seemed to compensate for reduced cholesterol by increasing cholesterol uptake from the circulation because mRNA and protein levels of SR-BI and LDLR were significantly elevated in GF mice (Fig. 7A,B). ABCG5 and ABCG8 expression levels were somehow upregulated in GF mice, (Fig. 7A,B), despite that they are also targets of LXR^25^.

Consistently with intestinal changes in bile acids-related genes, Hepatic FXR mRNA was downregulated, and the mRNAs for cholesterol 7α-hydroxylase (CYP7A1) and 25-hydroxycholesterol 7α-hydroxylase (CYP7B1) were upregulated in GF mice. Hepatic CYP7A1 protein was also increased (Fig. 8B). Collectively, changes in intestinal and hepatic expression levels of bile acid metabolism-related genes imply a reduced enterohepatic recirculation of bile acids in GF mice.

## Discussion

We have previously shown that mice lacking NPC1L1, the molecular target of ezetimibe^14^,^15^,^16^, have reduced stool output^12^. In this study, we demonstrate that ezetimibe reduces stool output in a microbiota-dependent manner and the gut microbiome is substantially altered in NPC1L1-deficient mice. In addition, we show that GF relative to SPF mice display reduced intestinal cholesterol absorption and increased fecal cholesterol excretion. These changes in cholesterol balance are translated into reduced plasma and hepatic levels of lipids. Intestinal gene expression analysis suggests that the negative balance of cholesterol in GF mice may partly be attributable to reduced NPC1L1 and increased ABCG5/ABCG8 in small intestine. Other gene expression alterations in small intestine and liver largely reflect reduced availability of cholesterol in these two tissues. Overall GF versus SPF mice are more sensitive to ezetimibe in lowering plasma and hepatic cholesterol. Our results together suggest that inhibiting microbiota may lower blood cholesterol, particularly when it is combined with a cholesterol-lowering drug such as ezetimibe.

Ezetimibe is an inhibitor of intestinal cholesterol absorption and it may also promote biliary cholesterol excretion in humans^18^. No studies have examined the effects of ezetimibe on cholesterol homeostasis in the absence of microbiota. This is an important question because ezetimibe may be coadministered with antibiotics and/or probiotics clinically. Here we observed a much greater effect of ezetimibe on almost all lipid parameters examined in GF mice than SPF mice. It is unlikely this effect was due to increased intake of ezetimibe that was mixed with our Western diet, because GF versus conventional mice were shown to consume similar amounts of Western diet^3^. While our results may suggest that ezetimibe is more effective when microbiota is absent, it remains possible that lack of microbiota promotes intestinal absorption of ezetimibe, thereby causing a greater effect. Detailed pharmacokinetic studies are required to address this issue in the future.

In an early study focusing on insulin resistance, Rabot and associate have fed GF and conventional mice a high-fat (60% energy) low-cholesterol (0.03%, w/w) diet^9^. This HFD is different from the Western diet (~45% energy, 0.2% cholesterol) used in this study. We chose a Western diet because its composition is similar to typical diets Western societies consume. While chow diets are regularly used in rodents, humans do not consume this type of diets. Nonetheless, GF mice on Rabot’s HFD also show altered cholesterol metabolism, as evidenced by reduced plasma cholesterol and triglycerides as well as increased fecal cholesterol excretion. They found that GF versus conventional mice have augmented activation of SREBP-2 and increased expression of HMG-CoA reductase in liver. In a recent study focused on regulation of bile acid metabolism by the gut microbiota, Sayin *et al.* also observed that GF mice have reduced levels of plasma cholesterol^11^. These findings, together with our data, collectively indicate that there is increased loss of cholesterol and a compensatory upregulation of mechanisms governing cholesterol synthesis in GF mice. Despite these similarities, Rabot and associate observed an increase in hepatic cholesterol while we found a decrease. This discrepancy is likely related to differences in diet compositions and diet feeding durations. In our studies, increased hepatic mRNAs for cholesterologenic genes may reflect a decrease in hepatic free cholesterol, especially in ezetimibe-treated groups. Rabot *et al.* did not examine intestinal cholesterol absorption, biliary lipid concentrations, intestinal mRNA expression, and protein expression of genes relevant to transport/metabolism of cholesterol and bile acids in small intestine and liver in their GF mice. Here we show for the first time that lack of microbiota reduces intestinal cholesterol absorption, at least in part, by inhibiting intestinal NPC1L1 expression, which, together with increased ABCG5/ABCG8 expression in small intestine, may explain why there is a substantial increase in fecal cholesterol excretion in GF mice. While biliary cholesterol is a significant source of fecal cholesterol, we found that GF mice relative to SPF mice have reduced biliary cholesterol concentration in the gallbladder bile, despite increased expression of ABCG5 and ABCG8, the heterodimeric canalicular cholesterol exporter^28^,^29^. A recent study also detected a decrease of biliary cholesterol in GF mice^11^. Therefore it is very unlikely that increased fecal cholesterol excretion resulted from changes in biliary lipid output in GF mice. Reduced biliary cholesterol levels in GF mice may result from decreased intestinal cholesterol absorption.

Overall, the gene expression pattern in liver and small intestine of GF mice is consistent with decreased uptake of cholesterol from the gut lumen and reduced delivery of cholesterol to liver except ABCG5 and ABCG8. These two proteins function as a heterodimer to transport cholesterol out from the apical side of enterocytes and hepatocytes^27^,^28^,^29^,^30^. It has been shown that ABCG5 and ABCG8 are targets of LXR^25^,^40^. In this case, one would expect ABCG5/ABCG8 expression to be downregulated in liver and intestine of GF mice due to reduced delivery of free cholesterol to these tissues. However, other transcriptional factors are also implicated in regulation of ABCG5/ABCG8 expression, including liver receptor homolog-1 (LRH-1), hepatocyte nuclear factor-4alpha (HNF-4α), and GATA^41^,^42^. While alterations in cellular levels of cholesterol and its intermediates can influence ABCG5/ABCG8 expression via the LXR-dependent mechanisms^43^, changes in bile acid metabolism and microbiota-derived factors may regulate expression of these gene at the same time through LRH-1, HNF-4 α, GATA or other unidentified mechanisms. A striking finding in this study is that GF mice have reduced expression of NPC1L1 in the jejunum. Transcriptional regulation of NPC1L1 expression is largely unknown^44^. Our observation suggests that intestinal NPC1L1 is subjected to microbiota regulation. Although the detailed molecular mechanism underlying this finding has yet to be defined, during evolution, microbiota-sustained NPC1L1 expression in small intestine may confer animals some advantage because this mechanism prevents excessive loss of cholesterol from the body. Alterations in expression levels of FXR, Ost-β, FGF15, Cyp7A and Cyp7B1 in small intestine and/or liver imply that GF mice also display substantial alterations in bile acid sensing, transport and metabolism. Consistently Sayin *et al.* has recently shown that the intestinal FGF15 expression is reduced and hepatic Cyp7A1 is increased in GF mice^11^. The gut microbiota is responsible for converting primary bile acids to secondary bile acids and it is not surprising to observe altered bile acid metabolism when microbiota is absent.

It was reported that gut Eubacterium coverts a proportion of cholesterol to coprostanol as its end product via an indirect pathway^45^. Since coprostanol is poorly absorbed and excreted as a neutral sterol in feces^46^, early studies tried to isolate gut bacteria that facilitate the conversion of cholesterol to coprostanol as a way to lower blood cholesterol^47^,^48^,^49^. However Rabot^9^ and we observed that GF mice have increased fecal neutral sterol excretion, suggesting that microbiota, as a community, prevents cholesterol loss from the body. These observations do not necessarily exclude the possibility that some species of gut bacteria, if expanded, will facilitate fecal cholesterol excretion. While increased fecal excretion and reduced intestinal absorption of cholesterol predict a protection against cholesterol-induced atherosclerosis, a study using conventional and GF apolipoprotein (apo) E knockout mice suggests that microbiota prevents atherosclerosis in mice on a regular chow diet and have no impact on atherosclerosis in mice on an extremely high (2%) cholesterol-containing diet^50^. It is difficult to compare these results with ours because our mice have intact apoE and are on a different diet. Our findings seem consistent with a couple of recent interesting studies. It was shown that trimethylamine-N-oxide (TMAO), a metabolite formed from substrates derived from the gut microbiota, promotes atherosclerosis^7^, and knockdown of the host enzyme flavin monooxygenase 3 (FMO3) that generates TMAO from bacterial substrates promotes macrophage reverse cholesterol transport in mice^51^.

Relative to research on how the gut microbiota regulates common metabolic disorders, few studies aimed at exploration of how host metabolism shapes the gut microbiome. Bile acids are catabolites of cholesterol. Recently it has been shown that bile acids substantially alter the gut microbiome^52^. Here we found that mice lacking NPC1L1, a cholesterol transporter that brings free cholesterol from the gut lumen into absorptive enterocytes, display increased Firmicutes and decreased Bacteroidetes, suggesting that the intestinal content of cholesterol has the potential to shape the gut microbiome. It was shown that obesity is associated with a decrease in Bacteroidetes and an increase in Firmicutes^53^,^54^, a change similar to that seen in L1-KO mice relative to wild-type control mice. However, L1-KO mice versus wild-type controls are protected against HFD-induced obesity and fatty liver^13^,^55^. Despite this, we are not surprised about our finding in L1-KO mice because inconsistent or opposite associations between the relative abundance of these bacteria and changes of body weight were also reported in other studies^56^,^57^,^58^,^59^. Altered gut microbiome warrants future studies focusing on definition of how cholesterol amounts in the gut lumen regulate the gut microbiome and how this regulation is related to cardio-metabolic disorders.

## Materials and Methods

### Statement of ethics of animal care and use

For the studies using L1-KO and control mice, all animal procedures were approved by the Institutional Animal Care and Use Committee at the University of Maryland. For the rest of animal studies, all animal protocols have been approved by the Animal Care and Use Committee of Southwest Hospital, the Third Military Medical University, China. All animal procedures were carried out in accordance with the U.S. National Institute of Health guidelines, including “Principles for Use of Animals” and “Guide for the Care and Use of Laboratory Animals”.

### Animals, diets and necropsy

Male SPF and age-matched GF C57BL/6J mice were provided by the Department of Laboratory Animal Sciences of the Third Military Medical University. GF mice were housed in polycarbonate cages containing sterile wood chips and the cages were maintained in sterile Trexler Plastic film isolators (Fengshi Laboratory Animal Equipment, China) at 23 ± 2 ^o^C and the relative air humidity of 50 ± 5% on a 12 h-light cycle. At the age of 8 weeks, all mice were randomly divided into 4 groups: SPF, SPF + ezetimibe (Ezet), GF, and GF + Ezet. SPF and GF group were fed a synthetic Western-type diet containing 17.3% protein, 48.5% carbohydrate (sucrose), 21.2% fat as lard, and 0.2% cholesterol. SPF + Ezet and GF + Ezet groups were fed the same Western diet mixed with ezetimibe at 0.07 mg/g. This content of ezetimibe was chosen based on the assumption that a 20 g mouse consuming ~3 g of the Western diet will be given ~10 mg/kg BW/day of ezetimibe, a common dosage used in mice^60^. The bedding and all diets were sterilized by 40 kGy Cobalt-60 Gamma irradiation in a facility at the Third Military Medical University. Water and bottles were high-pressure-steam sterilized at 121 ^o^C for 60 min. After 38 days of Western diet and ezetimibe treatments, the mice were fasted for 4 h (9AM–1PM during the daylight cycle) and then sacrificed for collection of blood, bile and tissues.

For 16S rRNA gene sequencing of the gut microbiome, L1-KO mice^61^ and their wild-type controls were fed a normal chow diet (Diet #: 2018, Harlan Teklad), or a HFD (D12492, Research Diet) for 5 weeks starting at 6 weeks of age. In the last week of diet feeding, the mice were individually housed and feces were collected for 72 h in the last 3 days of individual housing. Fecal samples were placed in ethanol and then sent to BGI Tech Solutions Co. Ltd, China for 16S rRNA gene pyrosequencing of the gut microbiome.

### Measurements of lipids in plasma, liver and bile

Plasma samples were analyzed for total cholesterol (Cat.#: E1005, Applygen, China), free cholesterol (Cat.#: E1006, Applygen, China), phospholipids (Cat.#: 296-63801, Wako, Japan) and triglycerides (Cat.#: E1003, Applygen, China) with enzymatic assay kits. For hepatic lipid contents, about 100 mg frozen liver sample from each mouse was used and lipids were extracted as described previously^62^. Extracted lipids were analyzed for total hepatic cholesterol (Cat.#: E1015, Applygen), free cholesterol (Cat.#: E1016, Applygen), triglycerides (Cat.#: E1013, Applygen) and phospholipids (Cat.#: 296-63801, Wako, Japan). For analysis of biliary lipid concentrations, a measured volume (5-10  μl) of gallbladder bile was extracted with chloroform: methanol (2:1) in the presence of 10 μg 5α-cholestane. The organic phase was analyzed for biliary cholesterol concentrations by gas chromatography and for phospholipid concentrations by a Phospholipids kit (Cat.#: 296-63801, Wako, Japan). The aqueous phase was used for biliary total bile acids by a Bile Acid kit (Cat.#: T047SC, Hermes Criterion Biotechnology, Canada).

### Measurements of fecal neutral sterol excretion

After 21 days of Western diet feeding and ezetimibe treatment, the mice were individually housed for fecal collection for 72 h. The feces were dried in a 70 ^o^C oven, and then weighed and crushed into powder. About 0.5 g of dried feces was put in a glass tube containing 5 ml of ethanol and 1 ml of 10N NaOH. The samples were digested at 120 ^o^C for 12 h, and then air-dried under 55 ^o^C in a heating block. After adding 7.5 ml of petroleum ether and 1.0 ml of 5α-cholestane as a standard in hexane (1 mg/ml), the tubes were shaken vigorously to mix samples and then centrifuged for 10 min at 1500 rpm. The hexane phase was transferred to a 2 ml gas chromatography vial. The neutral sterols were analyzed by gas chromatography. Fecal excretion of neutral sterols (cholesterol and its bacterial metabolites coprostanol and cholestanone) was calculated according to the following equation:

[(sterol area/5α-cholestane area)_sample_/(sterol area/5α-cholestane area)_standard_ x (amount of 5α-cholestane/g feces extracted) x (1000/387) x (100/g BW) = μmol/day/100 g BW].

### Measurements of intestinal cholesterol absorption

The dual fecal isotopic method was used^63^. After fecal collection for neutral sterol excretion, all individually housed mice were administered by gavage with 100  μl of soybean oil containing 1  μCi/ml [^14^C]cholesterol (DuPont) and 2 μCi/ml [^3^H]sitosterol (American Radiolabeled Chemicals, Inc., USA). The feces were collected for 3 days and homogenized in 10 ml 95% ethanol overnight, followed by lipid extraction with hexane and scintillation counting. Intestinal cholesterol absorption was then calculated by using the following equation: Percent cholesterol absorption=[(^14^C/^3^H ratio in dose − ^14^C/^3^H ratio in sample)/(^14^C/^3^H ratio in dose)] x 100.

### Gene expression analysis by quantitative real-time PCR (qPCR)

Total RNAs were collected from liver and the second proximal segment of small intestine that was equally divided into 5 segments in each mouse. The cDNAs were synthesized from total RNAs by using Quantscript RT kit for qPCR (Cat.#: KR103, Tiangen, China). DNA primers were synthesized by Sango Biotech (Shanghai) Co., Ltd. Sequences of primers are available upon request. The qPCR reaction was carried out in triplicate in a volume of 20 μl using SYBR Green PCR Master Mix (Cat.#: KK4609, KAPA Biosystems). GAPDH gene was used as an internal invariant control.

### Tissue protein expression analysis by Western blotting

About 40 mg of frozen liver and the entire middle segment of small intestine that was divided into 5 equal segments (~40 mg) were homogenized in RIPA buffer (50 mMTris-HCl, pH 8.0, 150 mMNaCl, 2 mM MgCl_2_, 0.1% SDS, 1.5% Nonidet P-40, and 0.5% deoxycholate) with protease inhibitors. The homogenate was centrifuged at 12,000 rpm for 20 min at 4 ^o^C. The total protein concentration of each sample was determined by a BCA protein assay kit (Pierce, Rockford, USA). From each sample, a total of 50 μg of total proteins was subjected to SDS-PAGE (8% for NPC1L1, 6% for ABCA1 and 10% for others) and Western blotting. Primary antibodies included a rabbit anti-mouse CYP7A1 antibody (Cat.#: ab65596, Abcam, UK), a rabbit anti-mouse/human LDL receptor antibody (Cat.#: ab52818, Abcam, UK), a mouse anti-ABCA1 antibody (Cat.#: ab18180, Abcam, UK), a rabbit anti-SR-BI antibody (Cat.#: ab52629, Abcam, UK), a rabbit anti-ABCG5 antibody (Cat.#: BS5013R, Bioss, China), and a goat anti-NPC1L1 antibody (Cat.#: ab121000, Abcam, UK).

### Fecal DNA extraction, bacterial 16S rRNA gene pyrosequencing, and bioinformatics analysis

Fecal DNA was extracted according to the methods of Zhang *et al.*^64^. The bacterial 16S rRNA genes were amplified with primers targeting the DNA region between the positions 27 and 1492 of Escherichia coli, corresponding to the V3-V5 region. The primers used were 27F/1492R (27F: 5’-AGAGTTTGATCMTGGCTC AG-3’, and 1492R: 5’-TACGGYTACCTTGTTACGACTT-3’). The pooled tagged single-stranded pyrosequencing library underwent fusion PCR and pyrosequencing in BGI Tech Solutions Co. Ltd (China) using Roche 454 FLX Pyrosequencer (Roche Life Sciences, USA) according to the manufacturer instructions. Experimental sequences were processed and analyzed using the Mothur platform ( http://www.mothur.org/wiki/Mothur_manual).

### Statistical analysis

All data were reported as Mean ± SEM (Standard Error of Mean). Significant differences were determined for the values among 4 groups by One-way ANOVA (Tukey-Kramer honestly significant difference). P value less than 0.05 was considered significant.

## Additional Information

**How to cite this article**: Zhong, C.-Y. *et al.* Microbiota prevents cholesterol loss from the body by regulating host gene expression in mice. *Sci. Rep.* **5**, 10512; doi: 10.1038/srep10512 (2015).

## Supplementary Material

Supplementary Information
