# Supplementary material for: Microbiota prevents cholesterol loss from the body by regulating host gene expression in mice
Source: Sci Rep. 2015 May 27;5:10512. doi: 10.1038/srep10512 (PMC4444975; doi:10.1038/srep10512)
Supplement: Supplementary Information [file srep10512-s1.pdf]

Supplemental Table

Microbiota prevents cholesterol loss from the body by regulating host gene expression in mice

Chun-Yan Zhong, Wei-Wei Sun, Yinyan Ma, Hongling Zhu, Pan Yang, Hong Wei, Ben-Hua Zeng, Qian Zhang, Yu Liu, Wen-Xia Li, Yixin Chen, Liqing Yu, and Zhi-Yuan Song

Table S1. Body and tissue weight of SPF and GF mice (g)

	SPF	GF	SPF+Ezet	GF+Ezet
BW	28.3±1.00[a]	26.03±1.27[b,c]	26.9±0.87[b]	24.39±1.05[d]
Liver weight	0.98±0.18[a]	0.86±0.08[b,c]	0.87±0.09[b]	0.76±0.09[d]
Liver/BW ratio (%)	3.48 ±0.63[a]	3.31+0.35[b]	3.23 ±0.33[b,c]	3.10 ±0.36[d]
SI weight	0.98±0.18[a]	0.73±0.07[c,d]	0.87±0.09[b]	0.76±0.07[c]
SI/BW ratio (%)	3.41 ±0.29[a]	2.78 ±0.22[c]	3.25 ±0.31[a,b]	2.71 ±0.20[c,d]
Ing.weight	1.47±0.01[a]	0.76±0.07[c]	1.16±0.08[b]	0.47±0.05[d]
Ing./BW ratio (%)	4.84 ±0.35[a]	2.90 ±0.19[c]	4.14 ±0.31[b]	1. 93 ±0.20[d]
EPI weight	1.61±0.15[a]	0.98±0.09[b]	0.82±0.11[c]	0.48 ± 0.08[d]
EPI/BW ratio (%)	5.06 ±0.52[a]	3.78 ±0.46[c]	4.15 ±0.38[b]	1.93 ±0.72[d]
BAT weight	0.42 ± 0.04[a]	0.41 ± 0.03[a]	0.38 ± 0.04[a]	0.39 ± 0.04[a]
BAT/BW ratio (%)	1.50 ±0.14[a]	1.57 ±0.10[a]	1.42 ±0.20[a]	1.60 ±0.20[a]

Eight-week-old SPF (n = 10-11) and GF (n = 10) mice were fed a Western diet for 38 days and then sacrificed for tissues. BW, body weight; SI, small intestine;

Ing., inguinal fat; EPI, epididymal fat; BAT, brown adipose tissue. The values with different small letters differ significantly (One-way ANOVA, $p < 0.05$).
